# Fucoidan Rescues *p*-Cresol-Induced Cellular Senescence in Mesenchymal Stem Cells via FAK-Akt-TWIST Axis

**DOI:** 10.3390/md16040121

**Published:** 2018-04-06

**Authors:** Jun Hee Lee, Chul Won Yun, Jin Hur, Sang Hun Lee

**Affiliations:** 1 Department of Pharmacology and Toxicology, University of Alabama at Birmingham School of Medicine, Birmingham, AL 35294, USA; j-school@hanmail.net; 2 Medical Science Research Institute, Soonchunhyang University Seoul Hospital, Seoul 04401, Korea; skydbs113@naver.com; 3 Departments of Biochemistry, Soonchunhyang University College of Medicine, Cheonan 330-930, Korea; 4 Center for Medical Innovation, Seoul National University Hospital, Seoul 03080, Korea; gene44@hanmail.net

**Keywords:** cellular senescence, chronic kidney disease, fucoidan, mesenchymal stem cells, *p*-cresol

## Abstract

Mesenchymal stem cells (MSCs) are a source for cell-based therapy. Although MSCs have the potential for tissue regeneration, their therapeutic efficacy is restricted by the uremic toxin, *p*-cresol, in chronic kidney disease (CKD). To address this issue, we investigated the effect of fucoidan, a marine sulfated polysaccharide, on cellular senescence in MSCs. After *p*-cresol exposure, MSC senescence was induced, as indicated by an increase in cell size and a decrease in proliferation capacity. Treatment of senescent MSCs with fucoidan significantly reversed this cellular senescence via regulation of SMP30 and p21, and increased proliferation through the regulation of cell cycle-associated proteins (CDK2, CDK4, cyclin D1, and cyclin E). These effects were dependent on FAK-Akt-TWIST signal transduction. In particular, fucoidan promoted the expression of cellular prion protein (PrP^C^), which resulted in the maintenance of cell expansion capacity in *p*-cresol-induced senescent MSCs. This protective effect of fucoidan on senescence-mediated inhibition of proliferation was dependent on the TWIST-PrP^C^ axis. In summary, this study shows that fucoidan protects against *p*-cresol-induced cellular senescence in MSCs through activation of the FAK-Akt-TWIST pathway and suggests that fucoidan could be used in conjunction with functional MSC-based therapies in the treatment of CKD.

## 1. Introduction

MSCs are a major source for stem cell-based regenerative medicine because of their self-renewing and multipotent capacity [1]. They can be isolated from several tissues such as adipose tissue, bone marrow, peripheral blood, and umbilical blood [2]. Transplanted MSCs have shown remarkable therapeutic efficacy in preclinical disease models in terms of their expansion and differentiation potential, secretion of cytokines and growth factors, and immunomodulatory effects [3]. However, the beneficial effects of MSCs in damaged tissues are restricted by pathophysiological conditions such as oxidative stress, limited nutrients, and ischemia, which result in death of the transplanted cells. Therefore, it is important to develop novel strategies to protect MSCs under pathophysiological conditions.

Chronic kidney disease (CKD) is a global public health problem due to a significant increase in hypertension, atherosclerosis, diabetes, and aging [4]. Kidney dysfunction leads to the excretion of toxic metabolites, commonly referred to as uremic toxins. These toxic products accumulate in the blood and have biochemical toxic effects in various tissues, which leads to complications such as cardiovascular disease and anemia, and neurological disorders [5]. *p*-cresol is one of the major uremic toxins. It is a protein-bound solute that induces apoptosis, cellular senescence, and causes an imbalance in the immune response [6]. Recent studies using animal models have suggested that stem cell-based therapies may have a regenerative effect in CKD, and that *p*-cresol may impair the functionality of mesenchymal stem cells (MSCs) [7]. Therefore, in order to apply MSC-based therapies to CKD patients, it is important to improve MSC functionality against uremic toxins.

Fucoidan, a marine sulfated polysaccharide extracted from brown algae and seaweed, has shown potential anticancer, antiviral, antibacterial, anti-inflammatory, and antioxidant effects [8]. In addition, fucoidan protects from kidney damage in streptozotocin-induced diabetic nephropathy [9]. Fucoidan also has shown beneficial effects in patients with liver disease caused by chronic hepatitis C virus infection [10]. Moreover, our previous investigations revealed that fucoidan can rescue cellular senescence in endothelial colony-forming cells [11], protect from oxidative stress in MSCs [12], and improve the functionalities of MSCs in a murine hind-limb ischemia associated with CKD [13]. However, although various studies have shown the beneficial effects of fucoidan in stem/progenitor cell and disease models, the protective effects of fucoidan on the uremic toxin, *p*-cresol, in MSCs has not been well investigated. 

Here, we investigated the protective potential of fucoidan on *p*-cresol-induced cellular senescence in MSCs via regulation of the focal adhesion kinase (FAK)-Akt-class A basic helix-loop-helix protein 38 (TWIST) axis. We found that modulation of the proliferation signaling pathway in *p*-cresol-induced senescent MSCs was regulated by fucoidan-mediated cellular prion protein (PrP^C^).

## 2. Results

### 2.1. Effects of p-Cresol on Cellular Senescence in MSCs

**Figure 1:**
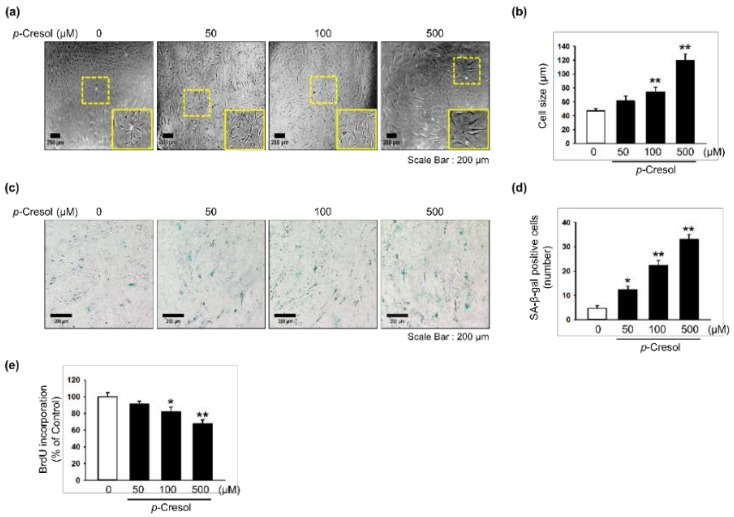
Effect of *p*-cresol on cellular senescence in MSCs. (**a**) Morphological changes in MSCs after treatment with *p*-cresol (50, 100, and 500 μM) for 72 h. Representative images are shown from one out of three independent experiments. Scale bar = 200 μm. (**b**) Determination of cell size (*n* = 10 images/cultured dishes). Values represent mean ± SEM. ** *p* < 0.01 vs. control. (**c**) After treatment with *p*-cresol (50, 100, and 500 μM) for 72 h, senescence was assessed by senescence-associated β-galactosidase (SA-β-gal) staining. SA-β-gal positive cells appear blue. Representative images are shown from one out of three independent experiments. Scale bar = 200 μm. (**d**) Cellular senescence was quantified as the number of SA-β-gal positive cells (*n* = 10 images/cultured dishes). Values represent mean ± SEM. * *p* < 0.05 and ** *p* < 0.01 vs. control. (**e**) After treatment with *p*-cresol (50, 100 and 500 μM) for 72 h, proliferation was assessed using a BrdU incorporation assay (*n* = 3). Values represent mean ± SEM. * *p* < 0.05 and ** *p* < 0.01 vs. control.

**Figure 2:**
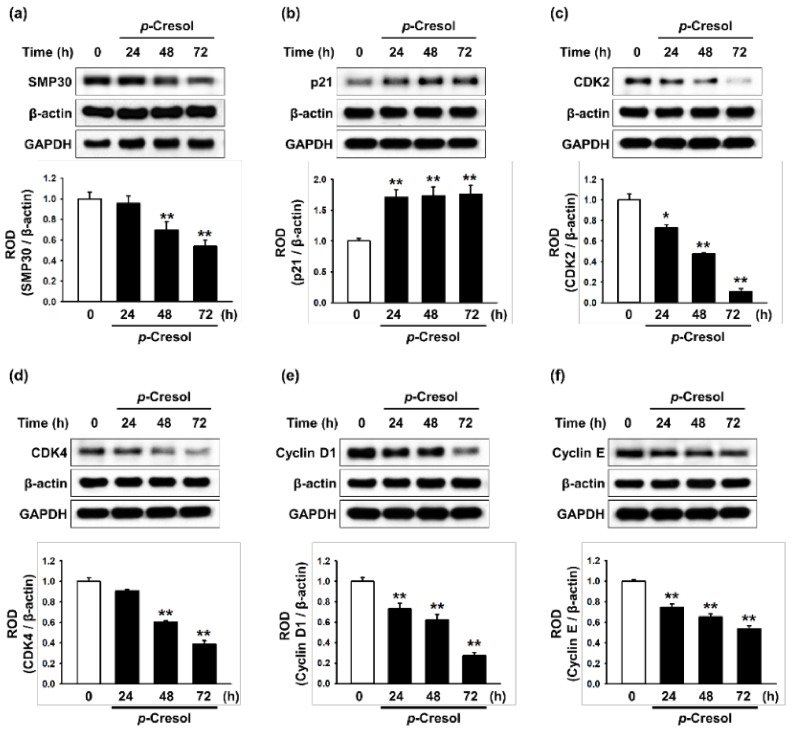
Effect of *p*-cresol on senescence- and cell cycle-associated protein expression in MSCs. (**a**–**f**) After treatment with *p*-cresol (500 μM) for 0, 24, 48 or 72 h, expression of SMP30 (**a**), p21 (**b**), CDK2 (**c**), CDK4 (**d**), cyclin D1 (**e**), and cyclin E (**f**) was assessed by western blotting. Protein levels were quantified by densitometry relative to β-actin levels (*n* = 3). Values represent mean ± SEM. * *p* < 0.05 and ** *p* < 0.01 vs. control.

To determine the effects of *p*-cresol on cellular senescence in MSCs, we assessed morphology, senescence, and proliferative capacity after treatment with *p*-cresol (50, 100, 500 μM) for 72 h. Treatment with *p*-cresol significantly increased cell size (Figure 1a,b). A senescence-associated β-galactosidase (SA-β-gal) assay showed that *p*-cresol treatment significantly increased senescence in MSCs (Figure 1c,d). In addition, a BrdU incorporation assay showed that treatment with *p*-cresol significantly decreased the proliferative capacity of the cells (Figure 1e), with 500 μM of *p*-cresol resulting in cell senescence. To further investigate the effect of *p*-cresol on senescence- and proliferation-associated signaling pathways in MSCs, the expression of senescence-associated proteins (SMP30 and p21) and cell cycle-associated proteins (cyclin-dependent kinase 2 (CDK2), CDK4, cyclin D1, and cyclin E) was assessed by western blotting. After treatment with *p*-cresol (500 μM) for 0, 24, 48, or 72 h, the expression of SMP30, an anti-senescence marker, was significantly decreased in a time-dependent manner (Figure 2a) and the expression of p21, a pro-senescence marker, was significantly increased (Figure 2b). The expression of CDK2, CDK4, cyclin D1, and cyclin E was also decreased in a time-dependent manner (Figure 2c–f). These results indicate that *p*-cresol induces MSC senescence and inhibits the proliferative potential of the cells.

### 2.2. Fucoidan Rescues Cellular Senescence Through the FAK-Akt-TWST Axis

**Figure 3:**
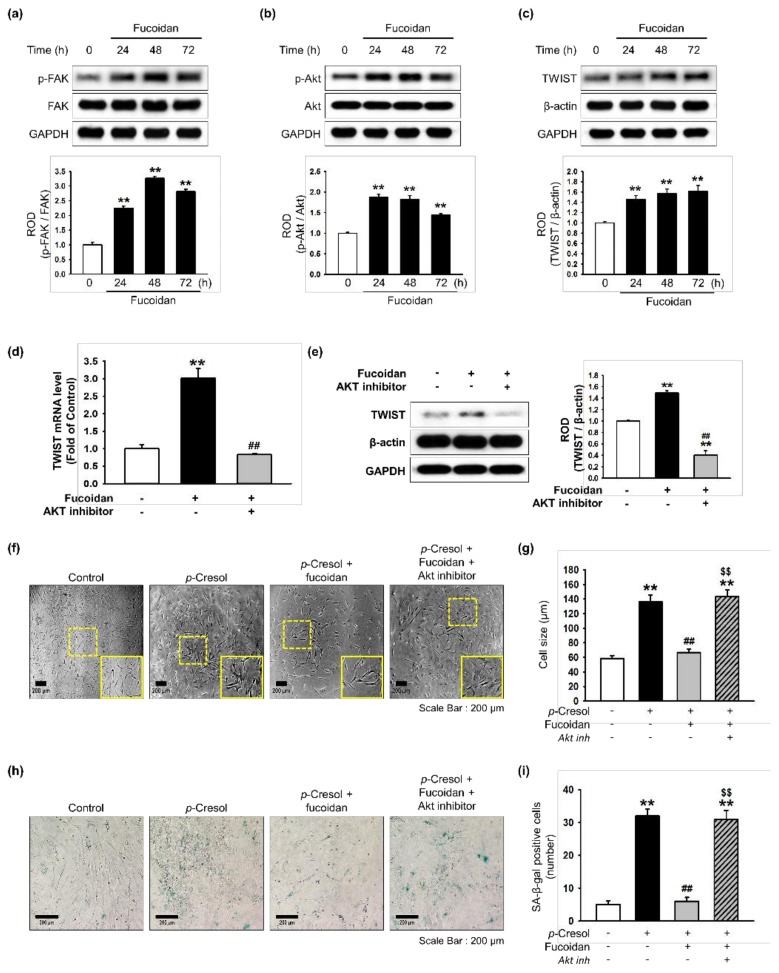
Effect of fucoidan on FAK-Akt-TWIST signaling in MSCs. (**a**–**c**) After treatment with fucoidan (10 μg/mL) for 0, 24, 48 or 72 h, expression of p-FAK (**a**), p-Akt (**b**), and TWIST (**c**) was assessed by western blot. Protein levels were quantified by densitometry relative to FAK, Akt and β-actin levels (*n* = 3). Values represent mean ± SEM. ** *p* < 0.01 vs. control. (**d**) MSCs were pretreated with an Akt inhibitor (10^−6^ M) for 30 min before treatment with fucoidan for 72 h; TWIST mRNA level was assessed by qPCR. (*n* = 3). Values represent mean ± SEM. ** *p* < 0.01 vs. control and ## *p* < 0.01 vs. treatment with fucoidan alone. (**e**) MSCs were pretreated with an Akt inhibitor (10^−6^ M) for 30 min before treatment with fucoidan for 72 h; then TWIST expression was assessed by western blotting. Expression of TWIST was quantified by densitometry relative to β-actin expression (*n* = 3). Values represent mean ± SEM. ** *p* < 0.01 vs. control, ## *p* < 0.01 vs. treatment with fucoidan alone. (**f**) Morphological changes after pretreatment with or without Akt inhibitor (10^−6^ M; 30 min) and fucoidan (10 μg/mL) in MSCs treated with *p*-cresol (500 μM; 72 h). Representative images are shown from one out of three independent experiments. Scale bar = 200 μm. (**g**) Determination of cell size (*n* = 10 images/cultured dishes). Values represent mean ± SEM. ** *p* < 0.01 vs. control, ## *p* < 0.01 vs. *p*-cresol alone, $$ *p* < 0.01 vs. fucoidan pretreatment with *p*-cresol. (**h**) Pretreatment with or without Akt inhibitor (10^−6^ M; 30 min) and fucoidan (10 μg/mL) in MSCs after treatment with *p*-cresol (500 μM; 72 h, senescence was assessed by senescence-associated β-galactosidase (SA-β-gal) staining. SA-β-gal positive cells appear blue. Representative images are shown from one out of three independent experiments. Scale bar = 200 μm. (**i**) Cellular senescence was quantified as the number of SA-β-gal positive cells (*n* = 10 images/cultured dishes). Values represent mean ± SEM. ** *p* < 0.01 vs. control, ## *p* < 0.01 vs. *p*-cresol only, $$ *p* < 0.01 vs. fucoidan pretreatment with *p*-cresol.

**Figure 4:**
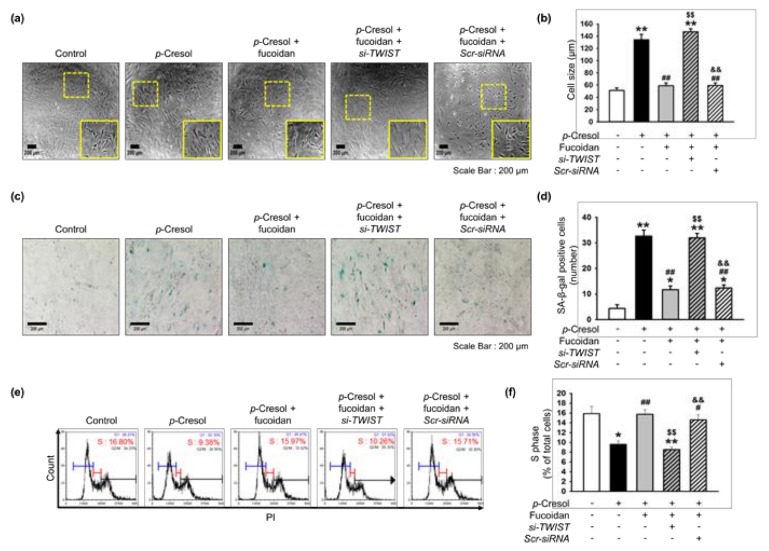
Fucoidan rescues *p*-cresol-induced cellular senescence in MSCs via the FAK-Akt-TWIST axis. (**a**) Morphological changes in MSCs, fucoidan-treated MSCs, and fucoidan-treated MSCs pretreated with TWIST siRNA (*si-TWIST*) after treatment with *p*-cresol (500 μM) for 72 h. Representative images are shown from one out of three independent experiments. Scale bar = 200 μm. (**b**) Determination of cell size (*n* = 10 images/cultured dishes). Values represent mean ± SEM. ** *p* < 0.01 vs. control (non-treatment), ## *p* < 0.01 vs. treatment with *p*-cresol alone (*p*-cresol), $$ *p* < 0.01 vs. treatment with *p*-cresol and fucoidan (*p*-cresol+fucoidan), && *p* < 0.01 vs. treatment with *p*-cresol, fucoidan, and *si-TWIST* (*p*-cresol+fucoidan+*si-TWIST*). (**c**) After *p*-cresol (500 μM, 72 h) exposure, senescence of MSCs, fucoidan-treated MSCs, and fucoidan-treated MSCs pretreated with *si-TWIST* was assessed by senescence-associated β-galactosidase (SA-β-gal) staining. SA-β-gal positive cells appear blue. Representative images are shown from one out of three independent experiments. Scale bar = 200 μm. (**d**) Cellular senescence was quantified as the number of SA-β-gal positive cells (*n* = 20 images/cultured dishes). Values represent mean ± SEM. * *p* < 0.05 and ** *p* < 0.01 vs. control, ## *p* < 0.01 vs. *p*-cresol, $$ *p* < 0.01 vs. *p*-cresol+fucoidan, && *p* < 0.01 vs. *p*-cresol+fucoidan+*si-TWIST*. (**e**) After *p*-cresol (500 μM, 72 h) exposure, the cell cycle was assessed in MSCs, fucoidan-treated MSCs, and fucoidan-treated MSCs pretreated with *si-TWIST* by flow cytometry with propidium iodide (PI) staining. (**f**) Proliferation capacity was quantified as the percentage of cells in the S phase (*n* = 3). Values represent mean ± SEM. * *p* < 0.05 or ** *p* < 0.01 vs. control, # *p* < 0.05 and ## *p* < 0.01 vs. *p*-cresol, $$ *p* < 0.01 vs. *p*-cresol+fucoidan, && *p* < 0.01 vs. *p*-cresol+fucoidan+*si-TWIST*.

Our previous study showed that TWIST is a key pathway involved in restoring cellular senescence in endothelial progenitor cells [14]. In addition, αMβ2 integrin is a major receptor for fucoidan and its activation leads to FAK, and the FAK-Akt signal transduction pathway is associated with protection against cellular senescence in endothelial colony-forming cells [11]. We determined that a concentration of 10 μg/mL of fucoidan most significantly inhibited cell senescence (Supplemental Figure S1). In addition, to explore whether fucoidan regulates the FAK-Akt-TWIST signal transduction pathway in time-dependent manner in MSCs, we evaluated activation of the FAK-Akt-TWIST signal pathway after treatment with fucoidan (10 μg/mL) for 0, 24, 48, or 72 h. As expected, phosphorylation of FAK and Akt was significantly increased after fucoidan treatment in a time-dependent manner, with the maximal effect seen at 48 h (Figure 3a,b). The expression of TWIST was also significantly increased in a time-dependent manner (Figure 3c). In addition, Akt inhibition blocked the mRNA and protein expression of TWIST, indicating that fucoidan-mediated TWIST expression is dependent on FAK-Akt phosphorylation (Figure 3d,e). Furthermore, our results show that the inhibition of Akt signaling increased the number of SA-β-gal positive cells and changed the cellular morphology of MSCs, suggesting that fucoidan-mediated Akt signaling is involved in the protection of *p*-cresol-induced MSC senescence (Figure 3f–i). To further investigate the protective effect of fucoidan on cellular senescence in MSCs via the FAK-Akt-TWIST axis, we assessed cellular senescence and proliferative potential in MSCs pretreated with fucoidan after exposure to *p*-cresol. Fucoidan treatment prevented the increase in cell size following *p*-cresol exposure (Figure 4a,b). In addition, a SA-β-gal assay showed that treatment with fucoidan significantly decreased MSC senescence caused by *p*-cresol exposure (Figure 4c,d). Furthermore, cell cycle analysis revealed that fucoidan inhibited the decrease in proliferation caused by *p*-cresol (Figure 4e,f). The protective effects against senescence were blocked by TWIST knockdown (Figure 4a–f). Taken together, these findings suggest that fucoidan protects MSCs against *p*-cresol-mediated cellular senescence via FAK-Akt-TWIST signal transduction.

### 2.3. Fucoidan-Mediated TWIST Expression Regulates Activation of Senescence- and Cell Cycle-Associated Proteins in MSCs after p-Cresol Exposure

**Figure 5:**
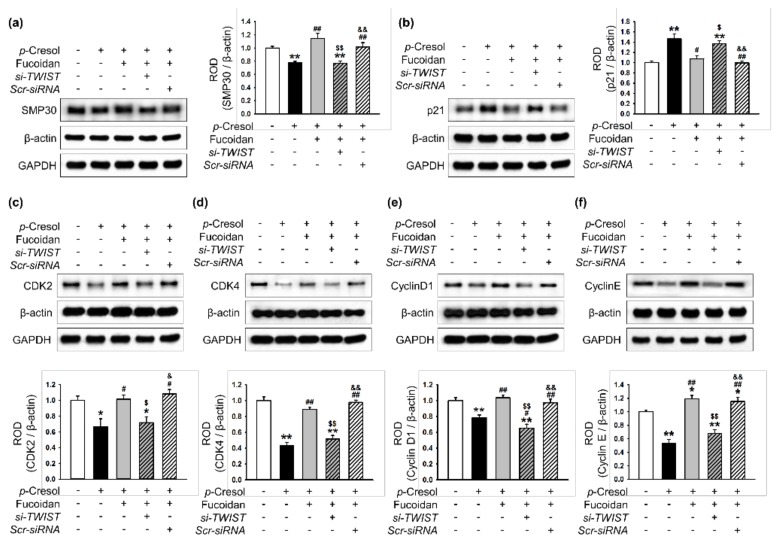
Regulation of senescence- and cell cycle-associated proteins in senescent MSCs is dependent on fucoidan-mediated TWIST expression. (**a**–**f**) After *p*-cresol (500 μM, 72 h) exposure, expression of SMP30 (**a**), p21 (**b**), CDK2 (**c**), CDK4 (**d**), cyclin D1 (**e**), and cyclin E (**f**) in MSCs, fucoidan-treated MSCs, and fucoidan-treated MSCs pretreated with *si-TWIST* was assessed by western blotting. Protein levels were quantified by densitometry relative to β-actin levels (*n* = 3). Values represent mean ± SEM. * *p* < 0.05 and ** *p* < 0.01 vs. control (non-treatment), # *p* < 0.05 and ## *p* < 0.01 vs. treatment with *p*-cresol alone, $ *p* < 0.05 and $$ *p* < 0.01 vs. treatment with *p*-cresol and fucoidan, & *p* < 0.05 and && *p* < 0.01 vs. treatment with *p*-cresol, fucoidan, and *si-TWIST*.

To investigate whether fucoidan-mediated TWIST expression regulates the activation of senescence-and proliferation-associated proteins after treatment with *p*-cresol, the expression of these proteins was assessed by western blotting. After *p*-cresol exposure, fucoidan inhibited the decrease in SMP30 expression and the increase in p21 expression (Figure 5a,b). Fucoidan also increased the expression of CDK2, CDK4, cyclin D1, and cyclin E (Figure 5c–f). Transfection with TWIST siRNA suppressed the protective effect of fucoidan on the regulation of senescence- and cell cycle-associated proteins (Figure 5a–f). These data indicate that fucoidan is involved in the regulation of senescence- and cell cycle-associated proteins in *p*-cresol-induced senescent MSCs, via the FAK-Akt-TWIST pathway.

### 2.4. Fucoidan Rescues p-Cresol-Mediated Inhibition of Proliferation in Senescent MSCs via the TWIST- PrP^C^ Axis

**Figure 6:**
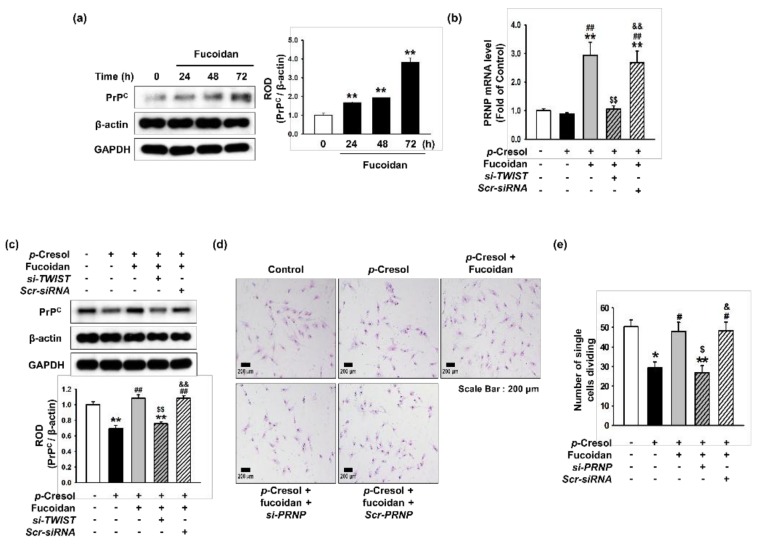
Effect of fucoidan on cell expansion in *p*-cresol-induced senescent MSCs through upregulation of PrP^C^ expression. (**a**) After treatment with fucoidan for 0, 24, 48, or 72 h, expression of PrP^C^ was assessed by western blotting. PrP^C^ levels were quantified by densitometry relative to β-actin levels (*n* = 3). Values represent mean ± SEM. ** *p* < 0.01 vs. control. (**b***) TWIST* siRNA- or *Scramble* siRNA-transfected MSCs pretreated with fucoidan (10 μg/mL) and *p*-cresol (500 μM; 72 h); then, *PRNP* mRNA level was assessed by qPCR. (*n* = 3). Values represent mean ± SEM. ** *p* < 0.01 vs. control, ## *p* < 0.01 vs. *p*-cresol alone, $$ *p* < 0.01 vs. Fucoidan-MSC with *p*-cresol, && *p* < 0.01 vs. Fucoidan-*si-TWIST* transfected MSCs with *p*-cresol. (**c**) After *p*-cresol (500 μM; 72 h) exposure, expression of PrP^C^ in MSCs, fucoidan-treated MSCs, and fucoidan-treated MSCs pretreated with *si-TWIST* was assessed by western blotting. PrP^C^ levels were quantified by densitometry relative to β-actin levels (*n* = 3). Values represent mean ± SEM. ** *p* < 0.01 vs. control (non-treatment), ## *p* < 0.01 vs. treatment with *p*-cresol alone, $$ *p* < 0.01 vs. treatment with *p*-cresol and fucoidan, && *p* < 0.01 vs. treatment with *p*-cresol, fucoidan, and *si-TWIST*. (**d**) After *p*-cresol (500 μM, 72 h) exposure, proliferative capacity in MSCs, fucoidan-treated MSCs, and fucoidan-treated MSCs pretreated with *si-PRNP* was assessed using a single cell expansion assay. Representative images are shown from one out of three independent experiments. Scale bar = 200 μm. (**e**) Single cell expansion capacity was quantified as the cell number (*n* = 10 images/cultured dishes). Values represent mean ± SEM. * *p* < 0.05 and ** *p* < 0.01 vs. control (non-treatment), # *p* < 0.05 vs. treatment with *p*-cresol alone, $ *p* < 0.05 vs. treatment with *p*-cresol and fucoidan, & *p* < 0.05 vs. treatment with *p*-cresol, fucoidan, and *si-PRNP*.

Several studies have revealed that PrP^C^ plays a pivotal role in stem cell expansion and self-renewal [15,16]. To determine whether fucoidan affects the expression of PrP^C^ in senescent MSCs, we treated normal MSCs with fucoidan for 0, 24, 48, or 72 h, and found that fucoidan induced PrP^C^ expression in a time-dependent manner (Figure 6a). After *p*-cresol exposure, the mRNA and protein expression of PrP^C^ was significantly inhibited; however, PrP^C^ expression was restored by treatment with fucoidan (Figure 6b,c). The effect of fucoidan was blocked by transfection with TWIST siRNA (Figure 6b,c). These results show that PrP^C^ expression decreases with cellular senescence, and that PrP^C^ expression is dependent on fucoidan-induced TWIST expression in senescent MSCs. To further explore whether fucoidan enhances the proliferative capacity of *p*-cresol-induced senescent MSCs, a single cell expansion assay was performed following *p*-cresol exposure. Treatment of MSCs with fucoidan prevented the *p*-cresol-mediated inhibition of self-renewal, and knockdown of PrP^C^ blocked the protective effect of fucoidan on proliferation (Figure 6d,e). Together, these findings suggest that the protective effect of fucoidan on proliferation in senescent MSC is mediated by the TWIST-PrP^C^ axis.

### 2.5. Fucoidan Enhances the Secretion of Angiogenic Cytokines and MSC Survival in Ischemic Tissues

**Figure 7:**
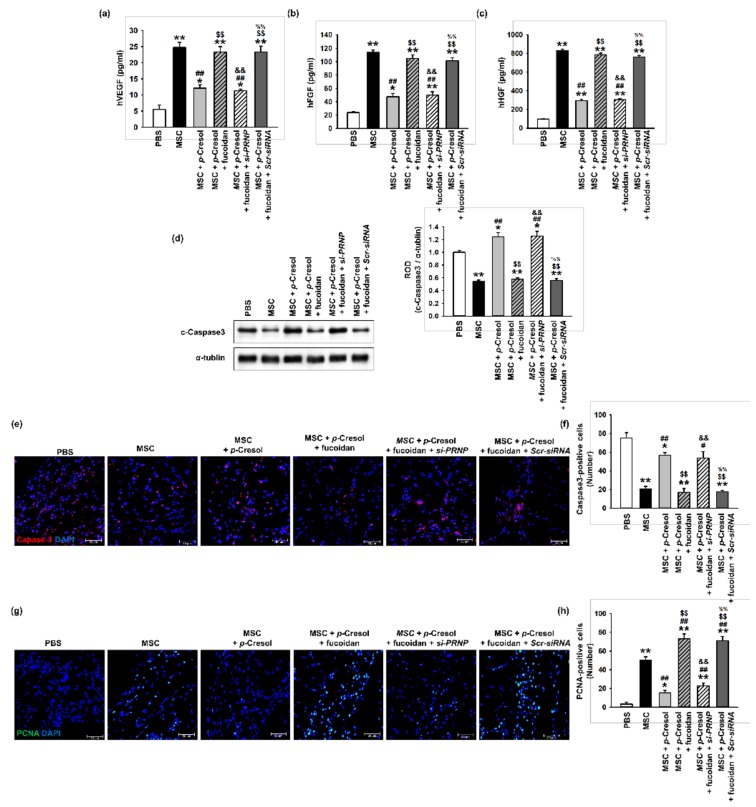
Effect of fucoidan on angiogenic cytokine expression and protection from apoptosis in a murine hindlimb ischemia model. (**a**–**c**) The expression of (**a**) hVEGF, (**b**) hFGF, and (**c**) hHGF in ischemic limb tissue lysates was determined by ELISA. Values represent the mean ± S.E.M. * *p* < 0.05 and ** *p* < 0.01 vs. PBS, ## *p* < 0.01 vs. MSC alone, $$ *p* < 0.01 vs. MSCs with *p*-cresol, && *p* < 0.01 vs. fucoidan pretreatment MSCs with *p*-cresol, %% *p* < 0.01 vs. fucoidan pretreatment *si-PRNP* transfected MSCs with *p*-cresol. (**d**) Western blot analysis of cleaved caspase-3 expression in ischemic sites of mice injected with PBS, MSCs, MSCs with *p*-cresol treatment, fucoidan-pretreated MSCs with *p*-cresol treatment, fucoidan-pretreated *si-PRNP*-transfected MSCs with *p*-cresol treatment, and fucoidan-pretreated *si-Scramble* transfection MSCs with *p*-cresol treatment at postoperative day 3. The level of cleaved caspase-3 was quantified by densitometry relative to α-tubulin levels (*n* = 3) Values represent mean ± SEM * *p* < 0.05 and ** *p* < 0.01 vs. PBS, ## *p* < 0.01 vs. MSC alone, $$ *p* < 0.01 vs. MSCs with *p*-cresol treatment, && *p* < 0.01 vs. fucoidan-pretreated MSCs with *p*-cresol treatment, %% *p* < 0.01 vs. fucoidan pretreated *si-PRNP*-transfected MSCs with *p*-cresol treatment. (**e**) At postoperative day 3, the level of apoptosis was evaluated by immunofluorescence staining for cleaved caspase-3 in ischemic limb tissues. Scale bar = 50 μm. (**f**) The levels of apoptosis were quantified as the number of cleaved caspase-3 positive cells. Values represent mean ± SEM. * *p* < 0.05 and ** *p* < 0.01 vs. PBS, # *p* < 0.05 and ## *p* < 0.01 vs. MSC alone, $$ *p* < 0.01 vs. MSCs with *p*-cresol treatment, && *p* < 0.01 vs. fucoidan-pretreated MSCs with *p*-cresol treatment, %% *p* < 0.01 vs. fucoidan pretreated *si-PRNP* transfected MSCs with *p*-cresol treatment. (**g**) At postoperative day 3, cell proliferation was evaluated by immunofluorescent staining for PCNA in ischemic limb tissues. Scale bar = 50 μm. (**h**) The levels of cell proliferation were quantified as the number of PCNA positive cells. Values represent mean ± SEM. * *p* < 0.05 and ** *p* < 0.01 vs. PBS, ## *p* < 0.01 vs. MSC alone, $$ *p* < 0.01 vs. MSCs with *p*-cresol treatment, && *p* < 0.01 vs. fucoidan-pretreated MSCs with *p*-cresol treatment, %% *p* < 0.01 vs. fucoidan-pretreated *si-PRNP* transfected MSCs with *p*-cresol treatment.

To determine whether fucoidan enhances the function and survival of MSCs exposed to uremic toxins, ischemic tissues were harvested at postoperative day 3 and a growth factor ELISA assay was performed (Figure 7a–c). The expression levels of angiogenic cytokines, including hVEGF, hFGF, and hHGF, were significantly increased following the injection of fucoidan-stimulated MSCs exposed to *p*-cresol, compared with those in the other groups. To investigate the survival of transplanted MSCs in ischemic damaged sites, the level of cleaved caspase-3 was analyzed in ischemic tissues at 3 days following MSC transplantation (Figure 7d). The expression of cleaved caspase-3 was significantly decreased following the injection of fucoidan-stimulated MSCs exposed to *p*-cresol (Figure 7d). In addition, we evaluated the viability and proliferation of transplanted MSCs into the ischemic site by immunofluorescence staining. The transplantation of fucoidan pretreated MSCs exposed to *p*-cresol significantly inhibited cleaved caspase-3 expression compared with non-pretreatment MSCs or PrP^C^-downregulated MSCs (Figure 7e,f). Furthermore, Proliferating cell nuclear antigen (PCNA) fluorescence staining demonstrated that cell proliferation in ischemic damaged tissues was significantly increased following the injection of fucoidan-treated MSCs exposed to *p*-cresol, compared with that in other groups (Figure 7g,h). These findings suggest that fucoidan protects MSCs from *p*-cresol-induced deficiencies in the secretion of angiogenic cytokines, cell survival, and proliferation following transplantation into ischemia sites via the regulation of PrP^C^ expression.

## 3. Discussion

Fucoidan, a sulfated polysaccharide found in brown algae and seaweed, has been shown to have a wide range of biological effects, including antitumor, antivirus, antithrombotic and anticoagulant, anti-inflammatory, antioxidant, and immunomodulation [17]. It has also shown protective effects against hepatic, renal, and uropathic disorders [17]. In particular, fucoidan contributes to the enhancement of cell functions such as adhesion, migration, proliferation, and differentiation by binding to adhesion proteins, growth factors, and cytokines [17]. Our previous studies revealed that fucoidan-stimulated endothelial colony-forming cells and MSCs were able to improve neovascularization and vascular regeneration in a murine hind-limb ischemia model by augmenting stem/progenitor cell bioactivities [11,12,13]. However, despite the beneficial effects of fucoidan, its effect on uremic toxins in stem/progenitor cells, and in particular, the underlying mechanism is still unclear. In the current study, we found that *p*-cresol, one of the major uremic toxins, induced cellular senescence in MSCs and that fucoidan treatment restored the deleterious effects of *p*-cresol on senescence and proliferation via the FAK-Akt-TWIST signal transduction pathway. Moreover, we showed that fucoidan-mediated PrP^C^ expression protected cells against the *p*-cresol-induced inhibition of proliferation via upregulation of TWIST expression. 

*p*-cresol is an end-product of tyrosine and phenylalanine catabolism. In patients with chronic kidney disease, *p*-cresol accumulates in the body because of decreased kidney function. This accumulated *p*-cresol, a uremic toxin, promotes oxidative stress and reduces the availability of NO, resulting in endothelial dysfunction and immune system imbalance in CKD [18]. *p*-cresol also promotes cellular senescence, and inhibits proliferation through cell cycle arrest [19,20,21]. In MSCs, *p*-cresol was shown to decrease the functionality and vitality of human bone marrow MSCs by inducing cell membrane damage [7]. Moreover, *p*-cresol can induce cellular senescence through the activation of autophagy [21]. Our results show that *p*-cresol induces senescence and inhibits proliferation in MSCs. *p*-cresol also increased the expression of the pro-senescence protein, p21, and decreased the expression of the anti-senescence protein, SMP30. The levels of cell cycle-associated proteins, CDK2, CDK4, cyclin D1, and cyclin E were also reduced. After *p*-cresol exposure, treatment with fucoidan protected MSCs against cellular senescence by regulating senescence- and proliferation-associated proteins. These findings indicate that fucoidan protects against *p*-cresol-induced cellular senescence through the regulation of senescence- and cell cycle-associated proteins.

Akt signaling is a well-known regulator of cell survival and proliferation [22]. Fucoidan is reported to have a high affinity for αMβ2 integrin [23], and our previous studies revealed that fucoidan activates FAK-Akt signaling in the enhancement of endothelial colony-forming cells and MSCs [11,12]. In addition, we found that Akt phosphorylation protects against cellular senescence through the downregulation of p21 and upregulation of SMP30 expression [11]. Moreover, fucoidan was shown to improve MSC proliferation and survival by protecting against oxidative stress via the Akt-MnSOD axis [12]. To further elucidate the exact mechanism of action of fucoidan, downstream of the FAK-Akt signal pathway, we focused on the role of TWIST signaling in recovering *p*-cresol-induced MSC senescence. Our results revealed that TWIST is downstream of the fucoidan-stimulated FAK-Akt signal pathway, and that fucoidan recovers the *p*-cresol-induced senescence of MSCs through FAK-Akt-TWIST-dependent signal transduction. TWIST is known to play a role in skeletal and mesodermal tissue development [24], and in cancer cells, TWIST induces metastasis, epithelial-mesenchymal transition, suppression of apoptosis, and generation of drug resistance [25]. Recent studies have shown that TWIST has important stem/progenitor cell properties [14,26], and overexpression enhances the maintenance and self-renewal of hematopoietic stem cells [26]. Senescent endothelial progenitor cells, because of replicative cultivation, can be recovered by activation of the hypoxia-inducible factor 1 alpha-dependent TWIST pathway [14]. Together, these findings suggest that fucoidan protects MSCs against *p*-cresol-induced cellular senescence through the FAK-Akt-TWIST axis.

Under pathophysiological conditions, the efficacy of MSC transplantation for regeneration is reduced owing to several risk factors such as oxidative stress, low nutrient levels, toxic metabolites, and inflammation. MSCs isolated from CKD mice showed reduced functionality, including a decrease in proliferation, secretion of pro-angiogenic cytokines, and neovascular formation [27]. In a murine hind-limb ischemia model associated with CKD, MSC transplantation did not significantly enhance functional recovery or neovessel formation [13]. PrP^C^, a highly conserved and ubiquitous glycoprotein, is an important molecule with roles in stem cell expansion and self-renewal [15,28]. PrP^C^ improves MSC ex vivo expansion and engraftment to bone marrow [29]. Furthermore, PrP^C^ expression facilitates the efficacy of MSC transplantation and neovascularization in a murine hind-limb ischemia model by enhancing their antioxidant and anti-inflammatory effects [16]. To understand the relationship between fucoidan and PrP^C^ in MSC proliferation after *p*-cresol exposure, we assessed PrP^C^ expression and MSC expansion capacity after *p*-cresol exposure and fucoidan treatment. Our data showed that fucoidan increased the expression of PrP^C^ and that its expression is dependent on the FAK-Akt-TWIST axis, resulting in augmentation of MSC expansion after *p*-cresol exposure. In addition, Fucoidan-pretreated MSCs increased growth factor secretion and cell survival rates after transplantation to ischemic site via the increased expression of PrP^C^. These findings suggest that fucoidan protects against the inhibition of *p*-cresol-induced self-renewal in MSCs via TWIST-dependent upregulation of PrP^C^ expression.

## 4. Materials and Methods

### 4.1. Cell Culture

MSCs derived from human adipose tissue were obtained from the American Type Culture Collection (ATCC; Manassas, VA, USA). MSCs were confirmed to be pathogen- and mycoplasma-free; they expressed cell surface markers such as cluster of differentiation (CD) 73 and CD105 but not CD31 and exhibited adipogenic and osteogenic differentiation potential when cultured in specific differentiation media. MSCs were cultured in α-minimum essential medium (GE Healthcare, Chicago, IL, USA) supplemented with 10% (*v*/*v*) fetal bovine serum (GE Healthcare), 100 U/mL penicillin (Thermo Fisher Scientific, Waltham, MA, USA), and 100 μg/mL streptomycin (Thermo Fisher Scientific). MSCs were cultured in a humidified incubator in an atmosphere of 95% air and 5% CO_2_ at 37 °C.

### 4.2. Preparation of Fucoidan

Fucoidan extracted from the seaweed *Fucus vesiculosus* was purchased from Sigma Sigma-Aldrich (St. Louis, MA, USA). Fucoidan was dissolved in phosphate buffered saline (PBS), filter-sterilized using a 0.45-μm pore filter, and stored as fucoidan extract at 4 °C until use.

### 4.3. Morphometric Analysis

Morphological changes in MSCs were examined by phase-contrast microscopy (Nikon, Tokyo, Japan). MSCs were cultured in 24-well plates (7000 cells/well). The size of adherent cells in a monolayer was calculated from images obtained using phase-contrast microscopy. Briefly, individual images of MSCs were obtained and the average cell size was calculated from a minimum of 3 field images per in 3 independent dishes using ImageJ software.

### 4.4. Senescence Associated β-Galactosidase Staining Assay

MSC senescence was determined from the percentage of cultured cells that were positive for SA-β-gal activity. MSCs were washed once with phosphate buffered saline (PBS) and fixed with 2% formaldehyde/0.2% glutaraldehyde (Sigma-Aldrich, St. Louis, MA, USA). Fixed cells were incubated at 37 °C for 24 h with β-gal staining solution (Sigma-Aldrich; 1 mg/mL X-Gal, 40 mM citric acid-sodium phosphate buffer, 150 mM NaCl, 2 mM MgCl_2_, 5 mM potassium ferrocyanide, and 5 mM potassium ferricyanide). Stained (blue, positive) and non-stained (negative) cells were counted using phase-contrast microscopy (Nikon), from three independent cultures.

### 4.5. Cell Proliferation Assay

Cell proliferation was assessed using a 6-bromo-2′-deoxyuridine (BrdU) incorporation assay. MSCs were cultured in 96-well plates (3000 cells/well). MSCs were exposed to *p*-cresol (500 μM) for 72 h. BrdU incorporation into newly synthesized DNA was assessed using an enzyme-linked immunosorbent assay (ELISA) colorimetric kit (Sigma-Aldrich). To perform the ELISA, 10 μM BrdU was added to the culture media and cells were incubated at 37 °C for 3 h. Cells were fixed with FixDenat solution and an anti-BrdU antibody (100 μL) was added to the cells, and incubated at room temperature for 90 min. Then, 100 μL substrate solution was added, followed by 1M H_2_SO_4_ to stop the reaction. Absorbance was measured at 450 nm using a microplate reader (BMG Labtech, Ortenberg, Germany).

### 4.6. Western Blot Analysis

Total protein was extracted from MSCs using RIPA lysis buffer (Thermo Fisher Scientific). Cell lysates and tissue homogenates (30 μg protein) in sample buffer were separated by electrophoresis on an 8–12% sodium dodecyl sulfate (SDS)-polyacrylamide gel and transferred to a polyvinylidene difluoride membrane for probing with antibodies. After washing with Tris-buffered saline/Tween-20 buffer (0.05% Tween-20, 150 mM NaCl, 10 mM Tris-HCl; pH 7.6), membranes were blocked with 5% bovine serum albumin for 1 h at room temperature then incubated with primary antibodies against SMP30, p21, CDK2, CDK4, Cyclin E, Cyclin D1, phospho-FAK, phospho-Akt, TWIST, PrP^C^, c-Caspase3, β-actin, and GAPDH (all from Santa Cruz Biotechnology, Santa Cruz, CA, USA). After incubation with peroxidase-conjugated secondary antibodies (Santa Cruz Biotechnology), bands were detected using enhanced chemiluminescence reagents (GE healthcare).

### 4.7. RNA Isolation and Quantitative Reverse Transcription Real Time PCR

Total RNA was extracted utilizing TRIzol^®^ reagent (Thermo Fisher Scientific). Reverse transcription polymerase chain reaction (RT-PCR) was performed utilizing RevertAid First Strand cDNA Synthesis Kit (Thermo Fisher Scientific) according to the manufacturer’s protocol. Quantitative real time PCR was performed utilizing the Rotor-Gene 6000 real-time thermal cycling system (Corbett Research, Mortlake, NSW, Australia) with a Maxima SYBR Green/ROX qPCR Master Mix (Thermo Fisher Scientific). PCR was performed under following cycling conditions; denaturation at 95 °C for 30 s, annealing at 60°o for 45 s, and extension at 72 °C for 45 s for 40 cycles. The data were analyzed by the comparative threshold cycle (CT) method and normalized against β-actin controls. Primer sequences were as follows: β-actin forward, 5′-AACCGCGAGAAGATGACC-3′; β-actin reverse, 5′-AGCAGCCGTGGCCATCTC-3′; TWIST forward, 5′-GTCCGCAGTCTTACGAGGAG-3′; TWIST reverse, 5′-CCAGCTTGAGGGTCTGAATC-3′; PRNP forward, 5′-ACAACTTTGTGCACGACTGC-3′; PRNP reverse, 5′- TGGAGAGGAGAAGAGGACCA-3′.

### 4.8. siRNA Transfection

MSCs were grown to 60–70% confluence in 100-mm culture plates and washed twice with PBS. MSCs were transfected for 48 h with SMART pool siRNAs (100 nM) specific to *TWIST, PRNP* mRNA, or control siRNA using Lipofectamine 2000 reagent (Thermo Fisher Scientific) in serum-free αMEM according to the manufacturer`s protocol.

### 4.9. Flow Cytometric Analysis for Cell Proliferation

MSCs treated with *p*-cresol, fucoidan, and *TWIST* siRNA were harvested, fixed in 70% ethanol at −20 °C for 48 h, and then washed twice with ice-cold PBS. Cells were incubated with RNase and propidium iodide (PI) for cell cycle analysis (SysmexPartec GmbH, Gorlitz, Germany). Cell cycle distribution was assessed using a Cyflow Cube 8 instrument (Partec, Munster, Germany). Data analysis was performed using standard FSC Express (De Novo Software, Los Angeles, CA, USA).

### 4.10. Single-Cell Cultivation Assay

A limiting dilution assay was used to aliquot single MSCs into individual wells of a 96-well culture plate. A cell suspension containing 1 × 10^3^ cells in 10 mL growth media was diluted tenfold, and 100 μL of the diluted sample (approximately 1 cell/100 μL) was seeded onto 96-well plates. Control MSCs and MSCs transfected with *PRNP* siRNA in the presence or absence of 500 μM *p*-cresol or 10 μg/mL fucoidan were cultured in a humidified incubator in an atmosphere of 95% air and 5% CO_2_ at 37 °C for 10 days.

### 4.11. Cell Transplantation in a Murine Hindlimb Ischemia Model

Cell transplantation experiments were performed on 8-week-old nude male BALB/c mice (Biogenomics, Seoul, Korea) maintained in a pathogen–free facility under a 12 h light/dark cycle at 25 °C with free access to water and regular laboratory chow, in accordance with National Research Council Guidelines for the Care and Use of Laboratory Animals. All animal procedures were approved by the Institutional Animal Care and Use Committee of Soonchumhyang University, Seoul Hospital, Korea (IACUC2013-5). Experiments utilizing the murine hindlimb ischemia model were performed as previously reported with minor modification. Briefly, ischemia was induced in mice by the ligation and excision of the proximal femoral artery and boundary vessels. No later than 6 hours after surgery, mice were injected intramuscularly into the ischemic thigh area (5 × 10^5^ cells/100 µL PBS per mouse; five mice per treatment group) with one of the following: PBS, untreated MSCs, *p*-cresol treated MSCs, *p*-cresol, and fucoidan-treated MSCs, *p*-cresol and fucoidan treated MSCs transfected with *PRNP* siRNA or scrambled siRNA. Cells were injected into five distinct ischemic sites.

### 4.12. Determination of Human Growth Factors

The levels of hVEGF, hFGF, and hHGF in the ischemic limb tissue (at 3 days post-surgery) lysates were determined by ELISA utilizing a commercially available ELISA kit (R&D Systems, Minneapolis, MN, USA) according to the manufacturer’s recommendation. All proteins were quantified using a bicinchoninic acid assay (BCA protein assay; Thermo Fisher Scientific). Growth factor expression levels were quantified by measuring the absorbance at 450 nm using a microplate reader (BMG Labtech).

### 4.13. Immunofluorescence Staining

Ischemic thigh areas were removed at 3 days post MSC transplantation, fixed with 4% paraformaldehyde (Affymetrix, Santa Clara, CA, USA), embedded in paraffin, and sectioned. For immunofluorescence staining, primary antibodies against cleaved caspase-3 or PCNA (Santa Cruz Biotechnology), and secondary antibodies conjugated to Alexa488 and Alexa594 (Thermo Fisher Scientific) were used. Nuclei were visualized by staining with 4′,6-diaminido-2-phenylindol (DAPI; Sigma-Aldrich). Immunostained slides were imaged by confocal microscopy (Olympus, Tokyo, Japan).

### 4.14. Statistical Analyses

Quantitative results are expressed as the mean ± SEM. All experimental results were analyzed by ANOVA. In some experiments, this was followed by a comparison of the treatment mean with the control using a Bonferroni-Dunn test. Data were considered significantly different at values of *p* < 0.05.

## 5. Conclusions

**Figure 8:**
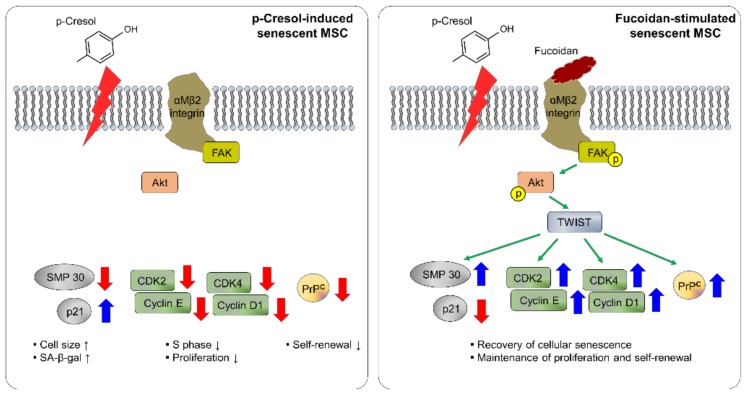
Schematic representation of possible mechanisms by which fucoidan protects against *p*-cresol-induced senescence in MSCs through the FAK-Akt-TWIST signal pathway. Fucoidan rescues the *p*-cresol-mediated inhibition of senescence- and cell cycle-associated proteins via FAK-Akt-TWIST signal transduction. In addition, fucoidan-mediated maintenance of proliferation is dependent on the TWIST-PrP^C^ axis. These effects of fucoidan protect against cellular senescence caused by *p*-cresol exposure in MSCs.

The protective effects of fucoidan on *p*-cresol-induced senescence in MSCs are summarized in Figure 8. Fucoidan protects against alterations in cell size, cell senescence, and inhibition of cell expansion by *p*-cresol. We show that the beneficial effects of fucoidan on cellular senescence caused by *p*-cresol are mediated by FAK-Akt-TWIST signal transduction. In particular, maintenance of proliferative capacity in MSCs is regulated by fucoidan-induced TWIST-dependent PrP^C^. In conclusion, our study suggests that fucoidan is a safe natural product that could be an effective stimulator for MSC-based therapeutics in CKD; thus, fucoidan-stimulated MSCs may be potential therapeutic agents for patients with CKD.

## Supplementary Materials

The following are available online at http://www.mdpi.com/1660-3397/16/4/121/s1. Figure S1: Effect of fucoidan inhibited the MSCs senescence by *p*-cresol.
