# Supplementary material for: Fucoidan Rescues p-Cresol-Induced Cellular Senescence in Mesenchymal Stem Cells via FAK-Akt-TWIST Axis
Source: Mar Drugs. 2018 Apr 6;16(4):121. doi: 10.3390/md16040121 (PMC5923408; doi:10.3390/md16040121)
Supplement: Supplementary file 1 [file marinedrugs-16-00121-s001.pdf]

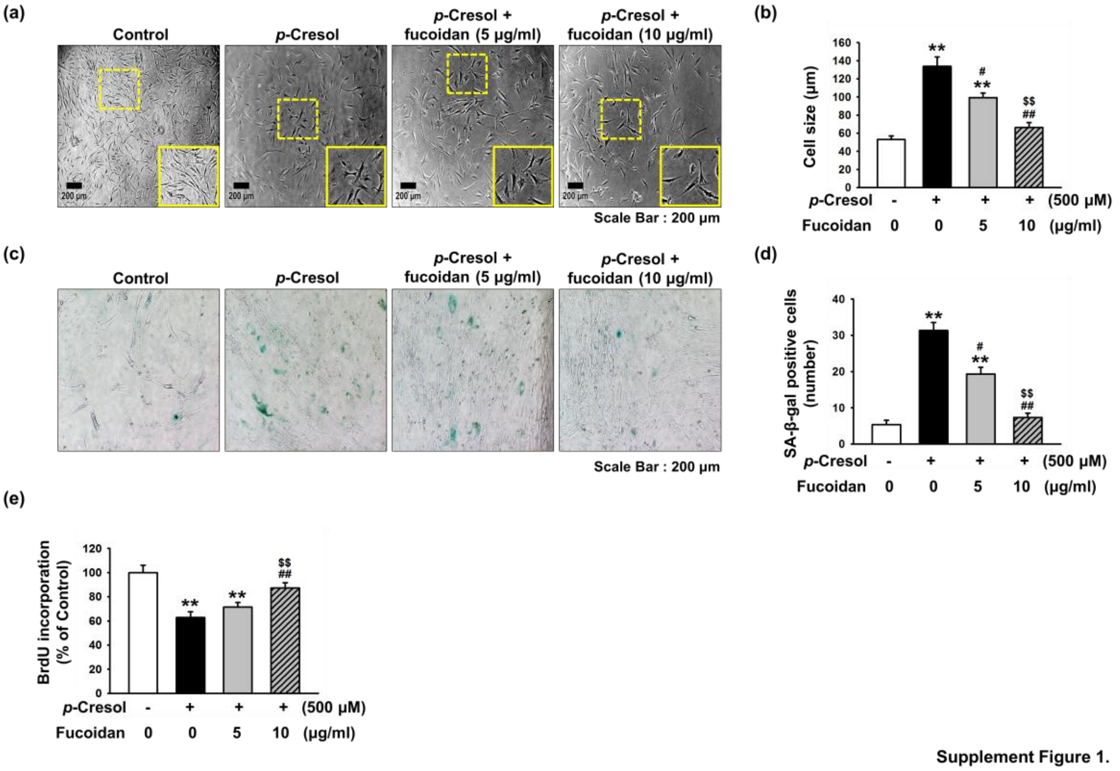

Supplemental Figure 1. (a) Morphological changes in fucoidan (5 or 10 μg/mL) pretreated MSCs after treatment with *p*-cresol (500 μM; 72 h). Representative images are shown from one out of three independent experiments. Scale bar = 200 μm. (b) Determination of cell size (n=10 images/cultured dishes). Values represent mean ± SEM. **p < 0.01 vs control, #p < 0.05 and ##p < 0.01 vs *p*-cresol alone, $$p < 0.01 vs fucoidan (5 μg/mL) pretreated MSCs after treatment with *p*-cresol. (c) Fucoidan (5 or 10 μg/mL) pretreated MSCs after treatment with *p*-cresol (500 μM; 72 h), senescence was assessed by senescence-associated β-galactosidase (SA-β-gal) staining. SA-β-gal positive cells appear blue. Representative images are shown from one out of three independent experiments. Scale bar = 200 μm. (d) Cellular senescence was quantified as the number of SA-β-gal positive cells (n=10 images/cultured dishes). Values represent mean ± SEM. **p < 0.01 vs control, #p < 0.05 and ##p < 0.01 vs *p*-cresol alone, $$p < 0.01 vs fucoidan (5 μg/mL) pretreated MSCs after treatment with *p*-cresol. (e) Cell proliferation was assessed using a BrdU incorporation assay (n=3). Values represent mean ± SEM. **p < 0.01 vs control, ##p < 0.01 vs *p*-cresol alone, $$p < 0.01 vs fucoidan (5 μg/mL) pretreated MSCs after treatment with *p*-cresol.
